# Alternol inhibits GAPDH activity and disrupts glycolytic flux preferentially in cancer cells

**DOI:** 10.3389/fphar.2026.1895065

**Published:** 2026-06-24

**Authors:** Yuelong Chai, Wang Liu, Moben Mirza, Hua Chen, Benyi Li

**Affiliations:** 1 Department of Plastic Surgery, The Affiliated Hospital, Guangdong Medical University, Zhanjiang, China; 2 Department of Urology, The University of Kansas Medical Center, Kansas City, KS, United States; 3 Department of Radiation & Medical Oncology, The Second Affiliated Hospital of Wenzhou Medical University, Wenzhou, China

**Keywords:** Alternol, cancer cells, GAPDH, glucose metabolism, glycolysis

## Abstract

**Introduction:**

We and others demonstrated that the natural compound Alternol induces apoptosis preferentially in human cancer cells. Glyceraldehyde-3-phosphate dehydrogenase (GAPDH) participates in cellular glycolysis, important for energy homeostasis, especially in cancer cells. We recently discovered that Alternol interacts with GAPDH, along with 4 Krebs cycle enzymes. In this study, we characterized the mechanism for Alternol-GAPDH interaction and the functional significance.

**Methods:**

Multiple human prostate cancer cell lines and a benign prostate epithelial cell line were utilized in the experiments. Enzyme activity assay *in vitro* with purified protein was used to examine Alternol inhibition of GAPDH activity. Computer-based docking assessment was performed to analyze Alternol interaction with GAPDH protein. Seahorse instrument was used to conduct glycolytic testing.

**Results:**

Our data revealed that Alternol interacts with GAPDH protein on two sites, one of which is the NAD^+^ binding site on the active domain of the enzyme, postulating an inhibitory effect. As expected, Alternol directly inhibited GAPDH dehydrogenase activity in an *in vitro* assay with purified enzyme with nanomole IC50 value at 5.794 nM. Consistently, Alternol significantly suppressed its enzymatic activity in cultured cancer cells but not in benign cells. These inhibitory effects were associated with reduced glycolytic capacity in cancer cells as assessed by extracellular acidification rate (ECAR) and metabolomic analysis.

**Conclusion:**

These results suggest that Alternol potently inhibits GAPDH activity and specifically disrupts glycolytic flux in cancer cells.

## Introduction

Metabolic reprogramming is one of the hallmarks in malignant cells and is characterized by the deregulation of metabolic pathways for the needs of rapid tumor growth and cancer cell proliferation (Hanahan and Weinberg, 2011). These altered metabolic pathways include increased uptake of glucose and amino acids and diverging metabolic intermediates from glycolysis/Krebs cycle to biosynthesis (Pavlova and Thompson, 2016). Among these metabolic reprogrammings, increased aerobic glycolysis is one of the main features in malignant cells (Krasnov et al., 2013).

Glycolysis is a relatively low-energy-producing pathway derived from an ancient metabolic process compared to mitochondrial respiration (Ganapathy-Kanniappan and Geschwind, 2013). However, due to limited nutrient supply and reduced oxygen concentration, cancer cells utilize metabolites derived from glycolytic flux to facilitate increased demands for macromolecule biosynthesis (Krasnov et al., 2013). Therefore, targeting the altered glycolysis pathway in cancer cells emerged as a novel approach for cancer therapy in recent years (Gill et al., 2016).

In mammalian cells, GAPDH exists as a homo-tetramer in cytoplasm compartment and acts as the essential component of the cellular glycolysis machinery (Krasnov et al., 2013). In the sixth step of glycolytic flux, GAPDH utilizes inorganic phosphate to produce organic bioenergy ATP, a critical step for cellular energy homeostasis (Sirover, 2018). In addition, GAPDH also represents a quintessential moonlight protein with different activities in multiple cellular compartments (Krasnov et al., 2013; Sirover, 2018). Although it was widely used as an internal control for gene expression studies, increased GAPDH expression was reported in multiple cancers with poor prognosis (Sirover, 2018; Zhang et al., 2015). Most interestingly, recent studies showed that GAPDH activity is a critical rate-limiting factor in cancer cells with high levels of aerobic glycolysis and that GAPDH inhibition with a small chemical compound koningic acid or oxidative stress dramatically affected glycolytic flux selectively in highly glycolytic cancer cells (Liberti et al., 2017; Yun et al., 2015).

Small chemical Alternol was isolated from fungi fermentation (Liu et al., 2007). We have demonstrated that Alternol exerts a strong anti-cancer effect by inducing oxidative stress-dependent apoptosis preferentially in malignant cells (Tang et al., 2014). We recently discovered that Alternol interacted with 14 cellular proteins, including GAPDH, along with 4 Krebs cycle enzymes, leading to disturbed Krebs cycle flux and reduced ATP level in cancer cells (Li et al., 2019). In this study, we characterized the Alternol engagement with GAPDH protein and assessed the significance of this engagement in cancer cells. Our data revealed that Alternol binds to GAPDH protein on the active domain and inhibits its dehydrogenase activity, resulting in a significant disruption of glycolytic flux in cancer cells.

## Materials and methods

### Cell culture, antibodies, and reagents

The origin and culture conditions for human cancer cell lines PC-3, C4-2B, 22RV1, and benign cell line BPH1 were described in our publication (Li et al., 2019). Alternol was obtained from Sungen Bioscience (Shantou, China) as reported (Tang et al., 2014). Chemodrug Docetaxel was obtained from Cayman Chemical (Ann Arbor, MI). Antibodies for GAPDH, poly (ADP-ribose) polymerase (PARP), histone H3, and Actin were obtained from Cell Signaling Technology (Danvers, MA). Secondary antibodies were obtained from Santa Cruz Biotech (Santa Cruz, CA).

### GAPDH enzymatic assay and glucose measurement

GAPDH dehydrogenase assay kit was purchased from BioVision (catalog #K680, Milpitas, CA) for cell-based enzymatic activity assay by following the manufacturer’s instructions. This assay kit uses glyceraldehyde-3-phosphate (1.0 mM) as substrate to be converted to 1,3-bisphosphate glycerate by GAPDH. This reaction yields Nicotinamide adenine dinucleotide-hydrogen (NADH) that will be captured by a developer chemical to form a colored product with a maximal absorption at 450 nm. For the *in vitro* Alternol inhibitory assay of GAPDH activity, purified active enzyme (0.5 μg/reaction) from rabbit muscle was used together with the assay system provided by the cell-based assay kit. Alternol was first dissolved in dimethyl sulfoxide (DMSO) and then further diluted in the assay buffer.

Glucose levels were measured using a glucose colorimetric detection kit (Invitrogen #EIAGLUC) according to the manufacturer’s manual.

### Cellular thermal stability assays (CETSA) and western blot assay

Cells exponentially grown in culture media were treated with the solvent or Alternol at the indicated drug concentration and duration. Cells were harvested in Radioimmunoprecipitation assay (RIPA) buffer for western blot or subjected to CETSA as described in our recent publication (Li et al., 2019; He et al., 2017). Nuclear and cytoplasm compartment preparation was described (Tsai et al., 2010) using the NE-PER^TM^ extraction kit obtained from ThermoFisher (catalog #78833) according to the manufacturer’s protocol. Western blot assay with sodium dodecyl sulfate-polyacrylamide gel electrophoresis (SDS-PAGE) approach was conducted as reported in our previous publication (Tang et al., 2014; Li et al., 2019; He et al., 2017).

### Alternol-GAPDH in-silicon docking analysis

Protein-ligand *in silico* docking analysis was conducted as described in our recent reports (Li et al., 2019). Briefly, the published crystal structure (1ZNQ) (Ismail and Park, 2005) for human liver GAPDH protein was extracted from the Research Collaboratory for Structural Bioinformatics Protein Data Bank. The crystal structure contains a bound ligand, NAD, that was removed prior to Alternol docking.

### Extracellular acidification rate (ECAR) assay

ECAR was measured in real-time using XF24 extracellular flux analyzer (Seahorse Bioscience, Billerica, MA) as described (Zhang and Zhang, 2019). Cells were seeded in XF24-well plates (3.0 x 10^5^ cells per well; n = 5 wells per group) and were incubated overnight at 37 °C, 5% CO_2,_ and treated with 5 or 10 µM of Alternol for 3 h. Cells were then subjected to a glycolysis stress test per the manufacturer’s guidelines. Protein concentration was used to adjust the ECAR after completion of the experiments.

### Metabolomic analysis

Metabolomic analysis was conducted using a gas chromatography-mass spectrometry (GC-MS) at the University of Utah Metabolomics core facility. Cells were seeded in a p100 dish overnight and then treated with the solvent or Alternol for 4 h. After harvesting, cells were resuspended in a cold 90% methanol solution containing the internal standard d^4^-succinic acid to give a final concentration of 80% methanol in the cell pellet. After quick vortexing and sonication for 5 min, cell lysis was incubated at −20 °C for 1 h, followed by centrifugation at 20,000 x g for 10 min at 4 °C. The supernatant was then transferred for metabolomic analysis. All GC-MS analysis was performed with an Agilent 7200 GC-MS QTOF and an Agilent 7693A automatic liquid sampler. Dried samples were suspended in 40 µL of a 40 mg/mL O-methoxylamine hydrochloride in dry pyridine and incubated for 1 hour at 37 °C in a sand bath. Data were collected, metabolites were identified, and their peak area was recorded using MassHunter Quant software (Agilent). Data statistical analysis was performed using MetaboAnalyst^R^ 2.0 (Chong et al., 2019).

### Statistical analysis

Quantitative data are presented as mean ± SEM (Standard Error of the Mean) from at least three independent experiments. Western blot data are shown from a representative experiment. Statistical analysis was performed using the SPSS 20.0 software (SPSS, Chicago, IL), and the significance was defined as the *p*-value is less than 0.05 in a Student’s t-test.

## Results

### Alternol interacts with GAPDH

**FIGURE 1:**
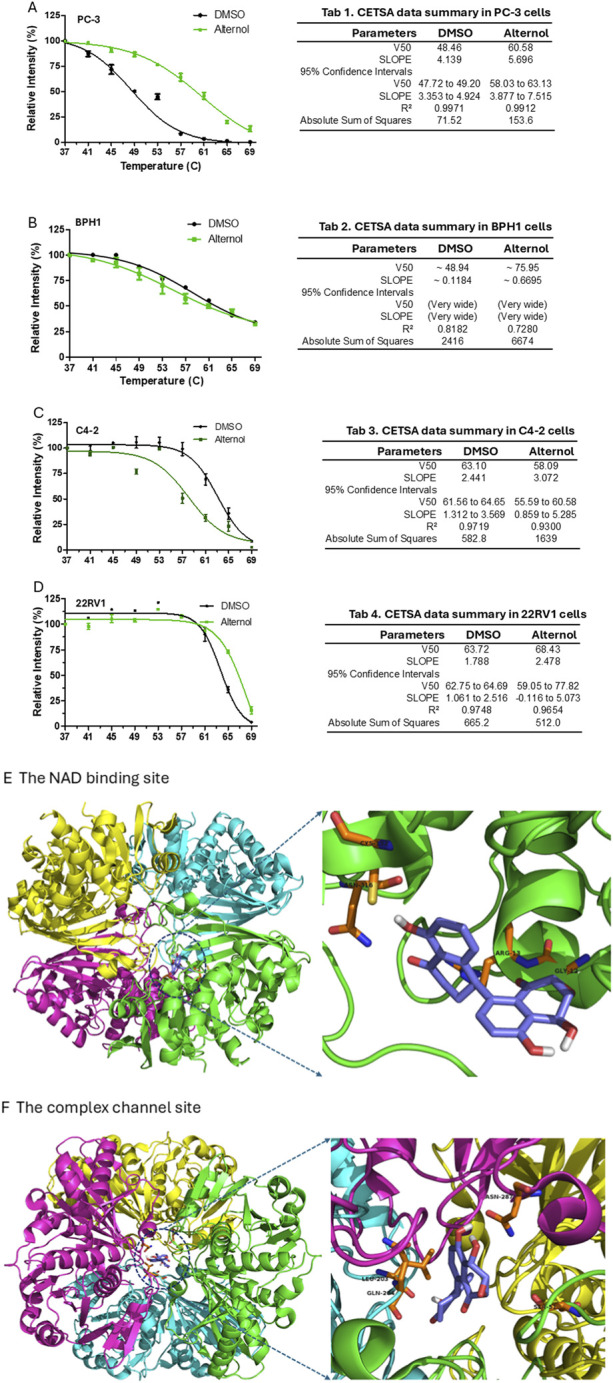
Alternol interacts with the GAPDH protein. **(A-D)** Cells were seeded in p100 dishes overnight and treated with DMSO or Alternol (10 μM) for 4 h. CETSA assay was performed as described (Li et al., 2019; He et al., 2017). Critical parameters were summarized in Tables 1-4. **(E-F)** The *in-silicon* docking analysis of Alternol-GAPDH interaction. The images show the cartoon view of the interaction site. Purple sticks indicate Alternol molecules, and orange sticks indicate amino acid residues that interact with Alternol. The right-hand panel shows the enlarged area of the dashed line circled area on the left panel.

We recently discovered that GAPDH is one of the 14 Alternol-interacting proteins in human cancer cells (Li et al., 2019). In this study, we first verified this ligand-protein engagement with a newly reported technique, the CETSA assay in a cell culture model (Martinez Molina et al., 2013). Cells were treated with Alternol for 3 h and then harvested for the assay. Alternol treatment dramatically shifted the elution curve of GAPDH protein to the right compared to the solvent control in PC-3, C4-2B, and 22RV1 cells (Figures 1A–C). The value of ΔT_m_50 is about 4 °C-12 °C, indicating a strong interaction. In contrast, there was no obvious separation in BPH1 cells (Figure 1D).

Second, to understand the molecular mechanism of Alternol-GAPDH interaction, we conducted an *in silico* docking analysis using the published crystal structure of human liver GAPDH protein (PDB#1ZNQ) (Ismail and Park, 2005). Alternol was found to bind strongly to GAPDH at two different sites, one corresponding to the catalytic active and NAD^+^ binding site on the amino acid residues G12, R13, N316, and C152 with a binding affinity at −10.1 kcal/mol (Figure 1E). Another site corresponds to a channel situated between the interface between all four GAPDH subunits on residues S51, N287, Q204, and L203 with a binding affinity at −9.4 kcal/mol (Figure 1F). These data suggest that Alternol might interfere with GAPDH catalytic activity since it binds to the GAPDH active site, competing with NAD^+^/substrates (Sirover, 2018).

### Alternol interferes with GAPDH catalytic activity

**FIGURE 2:**
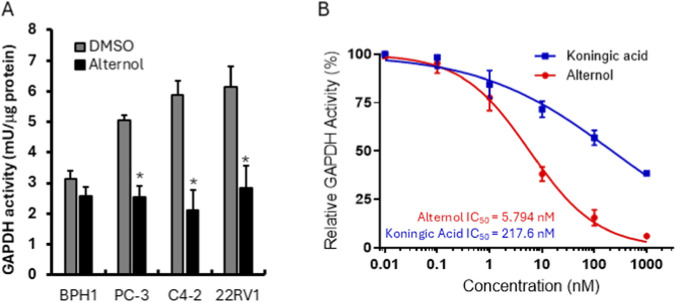
Alternol inhibits GAPDH activity. **(A)** Cells were seeded in 6-well plates and then treated with DMSO or Alternol (10 μM) for 3 h. GAPDH dehydrogenase activity was assessed as described in the text. GAPDH activity in each sample was normalized by its corresponding protein concentration. Data represents three independent experiments. The asterisk indicates a significant difference compared to the DMSO control (*p* < 0.05, ANOVA analysis). **(B)** Purified GAPDH enzyme from rabbit muscle preparation was utilized in the *in vitro* GAPDH activity assay as described in the text.

Then, we examined the effect of Alternol treatment on GAPDH catalytic activity in multiple human cancer cell lines. As shown in Figure 2A, GAPDH activity is relatively higher in malignant cells compared to benign BPH1 cells. However, Alternol treatment drastically reduced GAPDH activity in malignant cells but only a slight reduction in BPH1 cells. Since oxidative stress was reported to affect GAPDH activity (Yun et al., 2015) and Alternol caused reactive oxygen species (ROS) accumulation in cancer cells (Tang et al., 2014), we determined if Alternol-induced suppression of GAPDH catalytic activity was a primary or secondary event. An *in vitro* enzymatic assay was conducted with purified active GAPDH enzyme, and glyceraldehyde-3-phosphate was used as the substrate. As shown in Figure 2B, Alternol dramatically inhibited GAPDH dehydrogenase activity in a concentration-dependent manner with a half-maximal inhibitory concentration (IC_50_) at 5.794 nM. The 95% confidence interval (95%CI) was 1.501-20.84 nM. This IC_50_ was about 36-fold more sensitive than the commonly used GAPDH inhibitor koningic acid (KA) (Sakai et al., 1990). These data demonstrate that Alternol is a GAPDH inhibitor, possibly by blocking its active site.

### Alternol suppresses cellular glycolytic activity

**FIGURE 3:**
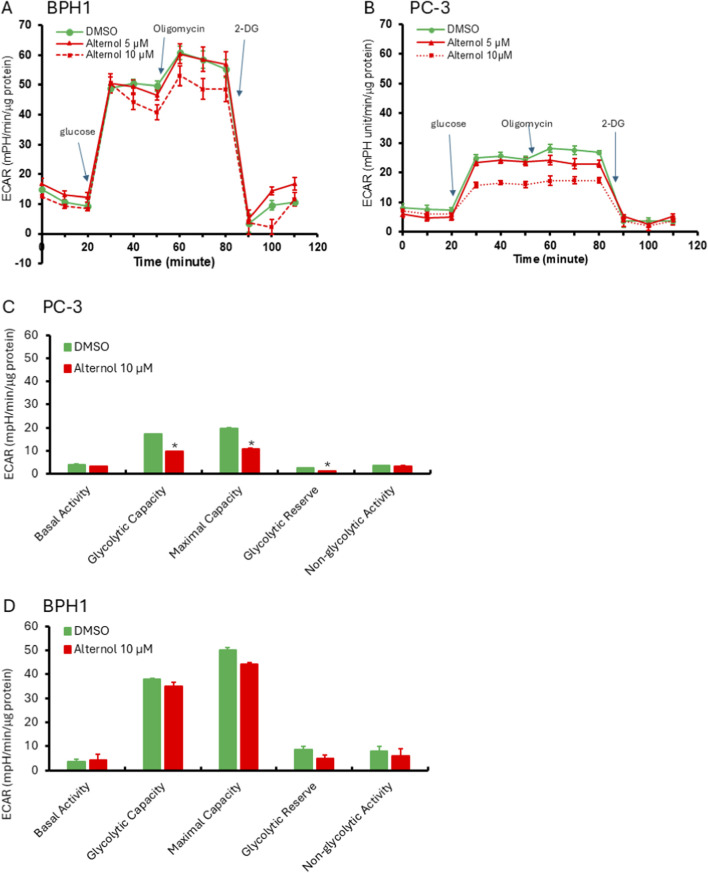
Alternol interferes with cellular ECAR in malignant cells. **(A,B)** Cells were seeded in the XF24 analyzer plates overnight and then treated with DMSO or Alternol (5-10 μM) during the experiments. Glycolysis stress protocol was utilized (chemical injection as arrow pointed) per the manufacturer’s instructions. The MEAN of ECAR readings from 5 repeats was shown at each time point. The error bar indicates SEM of the MEAN. **(C,D)** Glycolytic activity for each category was summarized as MEAN ± SEM. The asterisk indicates a significant difference compared to the DMSO control (*p* < 0.05, ANOVA analysis).

When glycolysis-derived end-product L-lactate is exported from the cell, protons are also exported, resulting in extracellular acidification (Mookerjee et al., 2015). Thus, extracellular acidification rate (ECAR) is an indicator for monitoring glycolysis. We analyzed ECAR in a pair of malignant and benign cells using the Seahorse XF analyzer system (Zhang and Zhang, 2019). Compared to benign BPH1 cells (Figure 3A), malignant PC-3 cells exerted a relatively lower glycolytic capacity, either in basal or stress (oligomycin treatment) conditions (Figure 3B). Alternol treatment significantly reduced glycolytic capacity in PC-3 cells (Figure 3C) but not in BPH1 cells (Figure 3D). These data suggest that Alternol interferes with cellular glycolytic capacity, possibly by inhibiting GAPDH catalytic activity.

### Alternol disrupts cellular glycolytic metabolism

**FIGURE 4:**
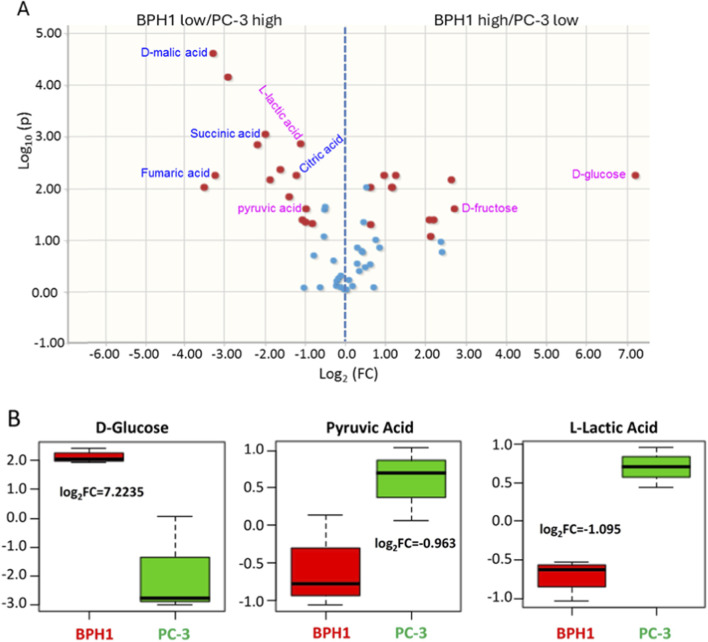
Metabolomic differences between malignant and benign cells. Exponentially grown PC-3 and BPH1 cells were harvested and subjected to metabolomic analysis with the GC-MS approach as described (Li et al., 2019). **(A)** The volcano plot was generated using the online tool Metaboanalyst for the comparison between BPH1 and PC-3 cells. Only significantly higher or lower glycolytic metabolites were labeled with pink font text. Previously reported Krebs cycle metabolites were labeled in blue font (Li et al., 2019). **(B)** Detailed comparison for individual metabolites was summarized, including glucose, pyruvate, and lactic acid.

Since glycolytic activities showed significant differences in response to Alternol treatment between BPH1 and PC-3 cells, as shown in Figure 3, we performed a deep analysis of glycolytic metabolomics using a gas chromatography-mass spectrometry (GC-MS) approach as described in our recent publication (Li et al., 2019). As shown in Figure 4A, a volcano plot showed the clear differences between BPH1 and PC-3 cells. As reported in our recent publication (Li et al., 2019), TCA metabolites (citric acid, malic acid, fumaric acid, and succinic acid) were significantly lower in BPH1 cells compared to PC-3 cells (blue font), reflecting a less active Krebs cycle in benign cells compared to malignant cells. For glycolytic metabolites, D-glucose level was significantly higher, but the end products of glycolysis (pyruvate and L-lactic acid) were significantly lower in BPH1 cells compared to PC-3 cells (Figure 4B). These data suggest that malignant cells possess a relatively higher glycolytic activity (in parallel with the Krebs cycle) compared to benign cells. Detailed comparisons of other metabolites are shown in Supplementary Table S1.

**FIGURE 5:**
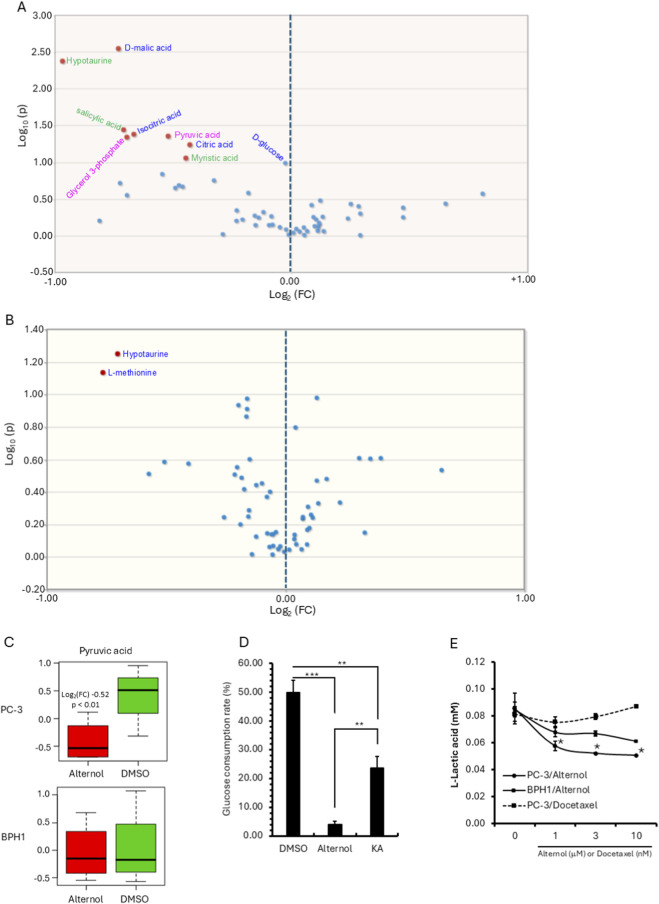
Alternol disrupts glycolytic flux in malignant but not benign cells. **(A,B)** Cells were treated with DMSO or Alternol (10 μM) for 4 h. Cells were harvested for the metabolomic analysis as described (Li et al., 2019). Alternol-induced alterations in metabolites in PC-3 **(A)** or BPH1 **(B)** cells were shown in the volcano plots. Glycolysis-related metabolites were labeled in pink font and non-related metabolites in green font. Previously reported Krebs cycle metabolites were labeled in blue font. **(C)** Pyruvic acid changes after drug treatment with Alternol in PC-3 and BPH1 cells. **(D)** PC-3 cells were treated with DMSO, Alternol (10 μM), or KA (10 µM) for 24 h under 10% FBS (FBS10) conditions. Glucose levels in cell media were measured after treatment. Consumption rate was calculated as: [(before-after)/before x 100%]. The asterisks indicate a significant difference (ANOVA analysis, **p < 0.01, ***p < 0.001). **(E)** Cells were treated with the compounds as indicated for 4 h. Cell culture media were collected for the measurement of L-lactic acid as described in the text. The asterisk indicates a significant difference compared to the control (ANOVA analysis, *p < 0.05).

Then, we analyzed the metabolomic alterations induced by Alternol treatment. In PC-3 cells, the most significant change was the reduced level of glycolytic end-product pyruvate after Alternol treatment (Figures 5A,C), along with previously reported Krebs cycle metabolites (D-malic acid, isocitric acid, and citric acid) (Li et al., 2019). However, these alterations were not observed in BPH1 cells (Figure 5B). These data indicate that Alternol alters glycolysis flux preferentially in malignant but not in benign cells. In addition, Alternol treatment in PC-3 cells also significantly reduced the level of glycerol 3-phosphate (Figure 5A), a metabolite at the intersection of glycolysis-lipid pathways.

Next, we compared the effect on glucose consumption between Alternol and koningic acid in PC-3 cells. As shown in Figure 5D, although both Alternol and KA significantly reduced cellular glucose consumption, Alternol showed a much more potent effect compared to KA. These data were in line with the *in vitro* GAPDH activity assay (Figure 2B).

Lastly, we assessed the changes in glycolytic end-product L-lactic acid after Alternol treatment. As shown in Figure 5E, Alternol treatment significantly reduced L-lactic acid levels in a concentration-dependent manner in PC-3 cells, while only a slight reduction was noticed in BPH1 cells. The chemodrug Docetaxel had no obvious effect on L-lactic acid level in PC-3 cells. These data were consistent with the reduced levels of pyruvate after Alternol treatment, indicating a disrupted glycolytic flux in malignant cells.

### GAPDH protein is not altered after alternol treatment

**FIGURE 6:**
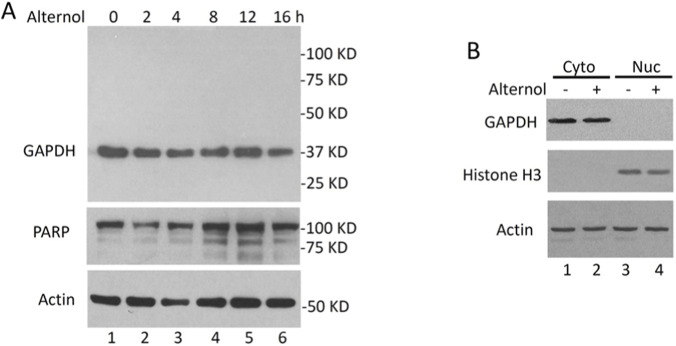
GAPDH protein expression after Alternol treatment. **(A)** PC-3 cells seeded in a p100 dish were left untreated or treated with Alternol (10 μM) for the indicated periods. A Western blot was conducted with the indicated antibodies. The Actin blot served as a protein loading control. **(B)** PC-3 cells were left untreated or treated with Alternol for 8 h. Cytoplasm and nuclear compartments were fractioned as described in the text. The Histone H3 blot was used as the protein loading control from the nuclear compartment preparation.

It was reported that oxidative stress induces GAPDH protein aggregation and nuclear translocation, leading to mitochondrial damage and apoptosis in neuronal cells (Nakajima et al., 2017; Dastoor and Dreyer, 2001). We assessed GAPDH protein levels after Alternol treatment. As shown in Figure 6A, Alternol induced PARP cleavage after 8 h treatment in PC-3 cells, a sign of apoptotic cell death that is consistent with our previous report (Tang et al., 2014). However, GAPDH protein appeared as a monomer band on SDS-PAGE gel after Alternol treatment, indicating protein aggregation is not involved in Alternol-induced GAPDH inhibition, which is different from a previous report (Nakajima et al., 2017). Meanwhile, GAPDH protein was not detected in nuclear compartment preparation after Alternol treatment (Figure 6B), which is not in line with previous reports (Dastoor and Dreyer, 2001). These data suggest that GAPDH nuclear translocation is not the main mechanism in Alternol-induced apoptosis in human cancer cells after Alternol treatment.

## Discussion

GAPDH is a soluble protein and physiologically participates in cellular glycolysis (Martin and Cerff, 2017). It is believed that GAPDH exerts non-glycolytic activities depending on the stimuli and its subcellular location (Tristan et al., 2011). During malignant transformation, the cellular metabolic program is altered to adapt to the cancerous microenvironment for survival and rapid growth. Among these altered metabolic programs, high levels of glycolytic activity are ubiquitously featured in solid tumors (Pavlova and Thompson, 2016). Furthermore, recent studies with computer modeling and metabolomic data mining showed that GAPDH-associated aerobic glycolysis is the most sensitive pathway for highly glycolytic cancer cells (Liberti et al., 2017; Shestov et al., 2014). Therefore, targeting GAPDH-dependent glycolysis emerged as a novel therapeutic approach in recent years (Krasnov et al., 2013; Ganapathy-Kanniappan, 2018).

In this study, we demonstrated that Alternol is engaged with GAPDH protein on its catalytic domain and directly inhibits its dehydrogenase activity at a nanomolar concentration. The IC_50_ value for Alternol on purified GAPDH enzyme was determined as 5.794 nM, which is highly effective (>36-fold) compared to the commonly used koningic acid with an IC_50_ value of 200 nM (Kato et al., 1992). Similar to the koningic acid (Sakai et al., 1991), Alternol also interacts with the GAPDH catalytic domain on C152/N316 residues with a high affinity (−10.1 kcal/mol). Although other compounds like 3-Bromopyruvate (Pereira da Silva et al., 2009), Iodoacetate (Nodin et al., 2012), Methylglyoxal (Lee et al., 2005), and Saframycin-A (Xing et al., 2004) were reported to inhibit GAPDH activity, what is lacking is the detailed molecular mechanism for ligand-protein interaction with IC_50_ values in the literature (Ganapathy-Kanniappan, 2018). Our data clearly defined Alternol as a potent GAPDH inhibitor stronger than koningic acid.

Glycolytic flux in malignant cells is excessively active due to deregulation of energy metabolism, which has been utilized for oncological diagnosis, such as positron emission tomography (PET) imaging (Lindenberg et al., 2019). It is conceivable that specifically blocking or suppressing this highly glycolytic flux will provide huge benefit in cancer management. Indeed, preclinical studies showed that disrupting glycolytic flux in cancer cells or xenografts achieved a remarkable anti-cancer outcome, and multiple enzymes involved in glycolytic flux were utilized as therapeutic targets, including GAPDH (Krasnov et al., 2013; Gill et al., 2016; Ganapathy-Kanniappan, 2018). Several small chemicals, such as 3-Bromopyruvate (Ko et al., 2019) and koningic acid (Kumagai et al., 2008), were used as GAPDH inhibitors for anti-cancer studies in preclinical models (Ganapathy-Kanniappan et al., 2012). In this study, our data revealed that Alternol disrupts glycolytic flux specifically in malignant but not benign cells, which provides a superb advantage over other small molecule GAPDH inhibitors for potential clinical development as an anti-cancer agent. Most significantly, Alternol also interacts with 4 major Krebs cycle enzymes and suppresses mitochondrial respiration, resulting in reduced mitochondrial oxygen consumption and cellular ATP production (Li et al., 2019). As the authors are aware, Alternol is the first compound reported in the literature that exerts an inhibitory effect on both glycolysis and mitochondrial respiration pathways in malignant cells but spares benign cells.

As a moonlight protein, GAPDH possesses various cellular activities in addition to its classic role in glycolysis. For example, GAPDH is reported to promote apoptosis under nitric oxide-mediated stress when it is S-nitrosylated on its active site, oligo-aggregated, and translocated into the nuclear compartment together with E3 ubiquitin ligase Sial1 in neuronal cells (Nakajima et al., 2017; Hara et al., 2005). In this study, our data showed that GAPDH appeared as a monomer on SDS-PAGE gel from cytoplasmic but not nuclear compartment preparation after Alternol treatment in human cancer cells, indicating a cell type- and stimuli-specific effect for its moonlighting function.

Taken together, our studies demonstrated that Alternol interacts with GAPDH, a metabolic enzyme critically involved in malignant transformation of cellular metabolism, and induces glycolytic disruption in malignant cells, consequently responsible for Alternol’s strong anti-cancer effect as evidenced in cell culture and mouse xenograft models (Tang et al., 2014; Li et al., 2019; Yeung et al., 2012).

## Data Availability

The datasets presented in this study can be found in online repositories. The names of the repository/repositories and accession number(s) can be found in the article/Supplementary Material.
